# Resident Astrocytes can Limit Injury to Developing Hippocampal Neurons upon THC Exposure

**DOI:** 10.1007/s11064-022-03836-1

**Published:** 2022-12-08

**Authors:** Maria Krassnitzer, Brooke Boisvert, Johannes Beiersdorf, Tibor Harkany, Erik Keimpema

**Affiliations:** 1 Department of Molecular Neurosciences, Center for Brain Research, Medical University of Vienna, Vienna, Austria; 2 Department of Neuroscience, Karolinska Institutet, Biomedicum 7D, Solna, Sweden

**Keywords:** Cell proliferation, Cannabis, Apoptosis, Astrocyte, Brain development

## Abstract

Cannabis legalization prompted the dilemma if plant-derived recreational drugs can have therapeutic potential and, consequently, how to address their regulation and safe distribution. In parallel, the steady worldwide decriminalization of cannabis and the enhanced content of its main psychoactive compound Δ^9^-tetrahydrocannabinol (THC), exposes populations to increasing amounts of cannabis and THC across all ages. While adverse effects of cannabis during critical stages of fetal neurodevelopment are investigated, these studies center on neurons alone. Thus, a gap of knowledge exists on how intercellular interactions between neighboring cell types, particularly astrocytes and neurons, could modify THC action. Here, we combine transcriptome analysis, transgenic models, high resolution microscopy and live cell imaging to demonstrate that hippocampal astrocytes accumulate in the strata radiatum and lacunosum moleculare of the CA1 subfield, containing particularly sensitive neurons to stressors, upon long term postnatal THC exposure in vivo. As this altered distribution is not dependent on cell proliferation, we propose that resident astrocytes accumulate in select areas to protect pyramidal neurons and their neurite extensions from pathological damage. Indeed, we could recapitulate the neuroprotective effect of astrocytes in vitro, as their physical presence significantly reduced the death of primary hippocampal neurons upon THC exposure (> 5 µM). Even so, astrocytes are also affected by a reduced metabolic readiness to stressors, as reflected by a downregulation of mitochondrial proteins. Thus, we find that astrocytes exert protective functions on local neurons during THC exposure, even though their mitochondrial electron transport chain is disrupted.

**Supplementary Information:**

The online version contains supplementary material available at 10.1007/s11064-022-03836-1.

## Introduction

In our current political climate, cannabis has taken the vanguard in discussions regarding policy decisions on the usage of plant-derived medical preparations for therapeutic treatment, as well as its public legalization as a recreational drug. Given these changes, our population is increasingly exposed to higher levels of its main psychoactive compound Δ^9^-tetrahydrocannabinol (THC), especially through selective breeding of potent cannabis strains, as well as novel efficient ways to deliver high doses of purified cannabinoids (vaping). Historically, lower strength cannabis has been considered relatively safe for adult human consumption [1], both recreationally and therapeutically. In contrast, current cannabis preparations can ramp up brain THC concentrations to the μM range [2]. It is therefore not unexpected that modern THC preparations are increasingly associated with the appearance of acute psychosis [3], greater risk of mental illness [4] and novel diseases such as cannabis hyperemesis syndrome [5]. Although these pathologies have been described in (young)-adult populations [6, 7], the long-term effects of THC on the pre-adolescent brain are only beginning to be elucidated [8]. Understanding THC effects in children is of vital importance because life-long brain plasticity is enriched by the precise wiring of neuronal networks up until 25 years of age [9]. However, neurons cannot work in isolation but are dependent on communication and metabolic support of astrocytes [10], which enrich in numbers well into adulthood. During brain development, astrocytes not only guide neurons to their final positions and instruct differentiation, but also promote postnatal synaptogenesis [11, 12], especially in relation to glutamatergic synapses [11, 13]. Therefore, we posit that exogenous stressors, such as THC, could inadvertently alter astrocyte availability to increase neuronal support [10] and ameliorate survival, differentiation and synaptogenesis.

We have recently demonstrated that postnatal [8] THC exposure negatively impacts the survival of cannabinoid type 1 receptor (CB1R)-expressing excitatory and inhibitory neurons, by affecting their membrane integrity, as well as mitochondrial metabolic readiness. As the pool of CB1R-containing neurons was unequally affected by THC, with excitatory neurons being more sensitive, we hypothesized that a local neuroprotective effect could buffer their demise, alike in models of stroke, aging, other neurotoxins and even Alzheimer’s disease [14–16], pathologies with a significant bioenergetic component [12]. When analyzing our proteomics data for astrocyte-specific molecular changes [8], we find a significant increase (245%) in excitatory amino acid transporter 2 (EAAT2) expression, suggesting the enhanced removal of extracellular glutamate as an example of innate neuroprotection [17]. By neuromorphology, we find that resident astrocytes particularly accumulate in the strata radiatum (SR) and lacunosum moleculare (SLM) of cornu amonis subfield 1 (CA1), both in short and long-term THC treatment paradigms. This astrocytic redistribution has been observed in neurodegenerative disease [14, 18], and is associated with neuroprotection limiting hyperexcitability through regulation of EAAT2, which is lacking in pathologies such as temporal lobe epilepsy [19, 20]. Thus, we posit that astrocytes amass in sensitive regions to safeguard excitatory neuronal cohorts and their neurite extensions from pathological damage [10]. These findings were recapitulated in vitro, as low numbers of astrocytes, co-cultured with primary neurons, can prevent neuronal cell death in the presence of micromolar concentrations of THC. However, THC significantly reduced mitochondrial protein levels in co-culture experiments, suggesting decreased metabolic functioning. In sum, we demonstrate that astrocytes provide a neuroprotective environment during THC exposure, important for the support of sensitive neurons in the event of toxic insult.

## Results

### Experimental Design

To reveal the impact of exogenous THC exposure on the developing postnatal hippocampus, we designed both short- and long-term treatment protocols to distinguish between acute and prolonged effects on astrocyte survival, proliferation and positioning (Fig. S1a, a_1_). To visualize astrocytes, we used immunohistochemistry in GLAST-Cre^ERT/+^::R26R^tdTomato/+^ reporter mice (JAX labs), in which astrocytes expressing the excitatory amino acid transporter 1 (EAAT1/GLAST) [21] produce tdTomato when primed with tamoxifen. The stability of EAAT1 protein levels in our previous dataset, in comparison to EAAT2, makes this model a suitable genetic strategy for the detection of astrocytes. We first treated postnatal (P) day 9 pups with synthetic THC (Dronabinol) or vehicle solution, as well as 25 mg/kg 4-OH tamixofen (to induce tdTomato expression), for 2 days before sacrificing and collecting brain tissues at P16. Second, we prolonged daily exposure to 6 days and collected tissues 2 days after the last exposure (at P16) to limit the presence of acute effects for this paradigm. A 5 mg/kg THC dose was chosen throughout as this corresponds to a typical low-medium strength single consumed cannabis preparation of ~ 0.4 mg/kg for humans [22].

### Cannabinoid Receptor Expression

**Fig. 1:**
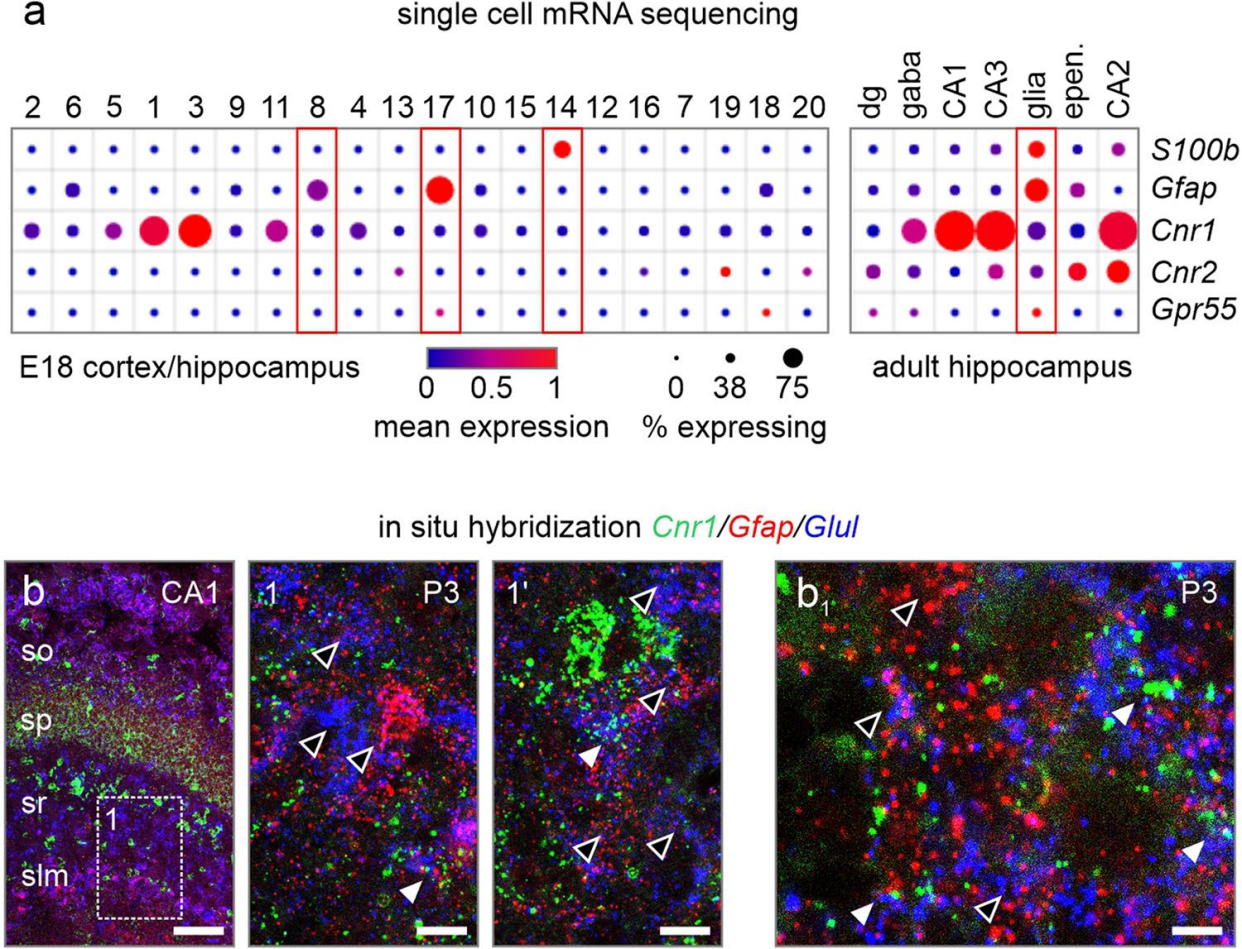
Expression of cannabinoid receptors in astrocytes. **a** Single cell mRNA analysis of E18 and adult hippocampal astrocytes [23, 26]. **b**, **b**_**1**_ In situ hybridization for *Cnr1* in P3 (**b**, **b**_**1**_) hippocampal tissues. Open arrowheads denote *Cnr1*-negative astrocytes, while closed arrowheads point to *Cnr1* expressing astrocytes. *CA1-3* cornu ammonis 1–3; *dg* dentate gyrus; *epen* ependymal cells. Scalebars = 100 µm (**b**), 25 µm (**1**, **1’**), 5 µm (**b**_**1**_)

We first verified if postnatal astrocytes possess receptors of the endogenous cannabinoid signaling system, through which THC mainly exerts its actions. In a previously published single-cell RNA-sequence dataset of (E18) cortex/hippocampus [23] cells, *Cnr1* was expressed in 5.63% and 7–10% in *S100b* and *Gfap*-containing astrocytes (Fig. 1a), respectively (analyzed through https://singlecell.broadinstitute.org/single_cell). Note that at this stage, neural stem cells also express *Gfap* [24, 25], making *S100b* a likely more specific marker of astrocytes. In the adult hippocampus [26], the percentage of *Cnr1*-expressing *S100b*^+^ astrocytes increased to 23.86%, indicating differentiation/development-related enrichment in *Cnr1* expression (Fig. 1a). In contrast, mRNAs for both *Cnr2* and *Gpr55*, a non-canonical cannabinoid receptor, were at the lower detection boundary (0.04 and 0.02% of total population), with *Cnr2* increasing into adulthood to 1.36% only and *Gpr55* remaining unchanged throughout. These observations suggest that *Cnr1* is the primary candidate to purvey THC-induced signaling cascades in postnatal astrocytes. In situ hybridization in both P3 and P8 hippocampi verified these findings by showing *Cnr1* expression in some *Gfap*^+^/glutamine synthetase (*Glul*^+^) astrocytes across the hippocampus, at levels significantly lower than those for neighboring pyramidal cells and interneurons (Fig. 1b, b_1_). Thus, hippocampal astrocytes are progressively poised to respond to cannabinoid signals through CB_1_Rs already at early postnatal life, at least in mice.

### Astrocyte Distribution and Cytoarchitectural Differentiation upon Postnatal THC Exposure

**Fig. 2:**
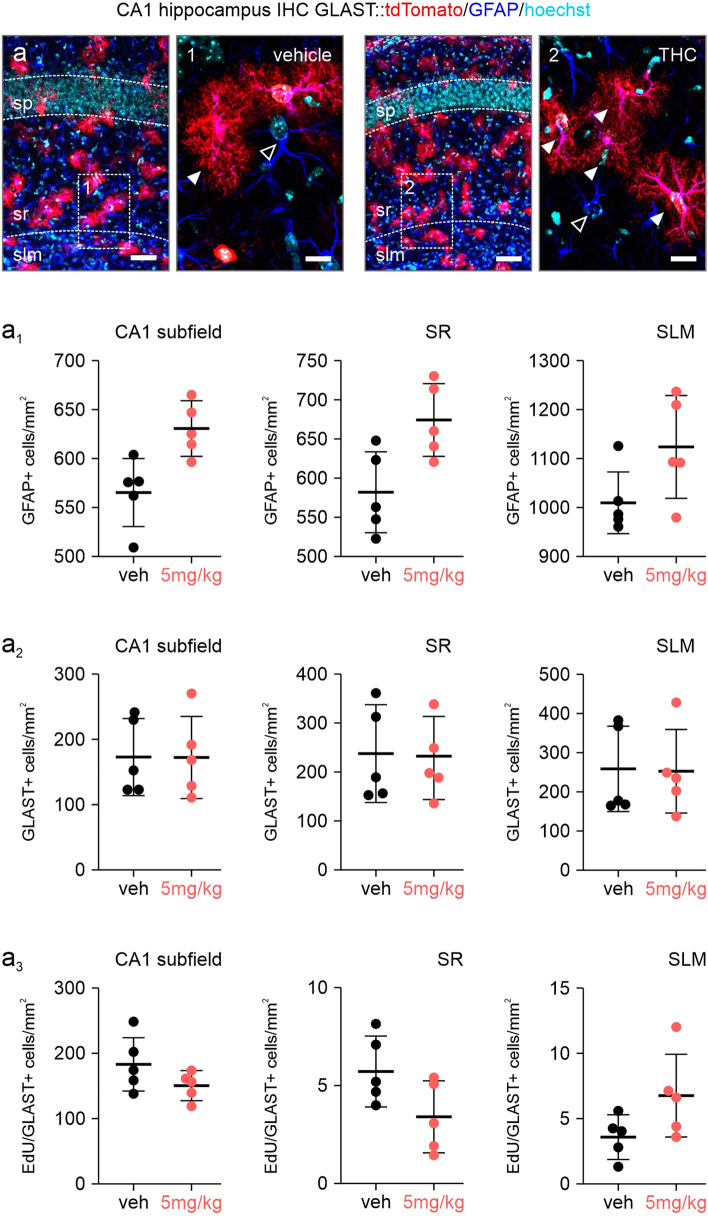
Astrocyte numbers after short term postnatal THC exposure. **a**–**a**_**3**_ Quantifications of GFAP (**a**_**1**_), GLAST (**a**_**2**_) and EdU-containing astrocytes (**a**_**3**_) in the stratum radiatum (SR) and stratum lacunosum moleculare (SLM) of the CA1 region of the hippocampus. Counts were expressed as cell numbers per mm^2^ tissue. Note the reduction of the stratum pyramidale (SP) thickness, reconfirming the loss of neurons previously reported [8]. Scalebars = 50 µm (**a**), 10 µm (**1**, **2**)

**Fig. 3:**
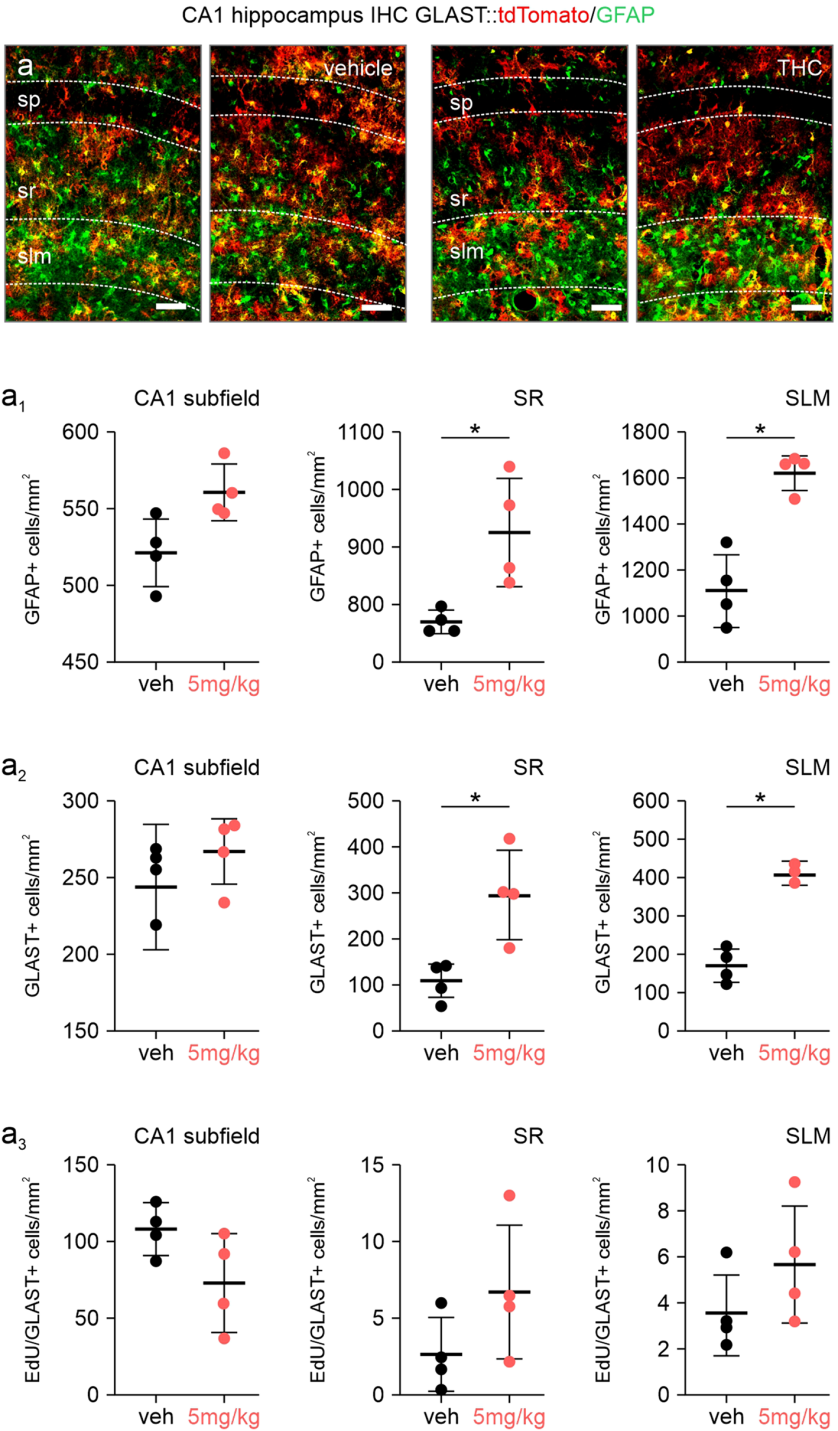
Astrocyte numbers after long term postnatal THC exposure. **a**–**a**_**3**_ Quantifications of GFAP (**a**_**1**_), GLAST (**a**_**2**_) and EdU-containing astrocytes (**a**_**3**_) in the stratum radiatum (SR) and stratum lacunosum moleculare (SLM) of the CA1 region of the hippocampus. Counts were expressed as cell numbers per mm^2^ tissue. Note the reduction of the stratum pyramidale (SP) thickness, reconfirming the loss of neurons previously reported [8]. Scalebars = 50 µm (a). *P ≤ 0.05

As the mitochondrial metabolic activity is negatively affected by THC through CB1Rs in adulthood [27], we sought to address if THC could affect the survival, positioning or morphology of hippocampal astrocytes in postnatal mice. Similar to our previous reported data, we first reconfirmed a reduction of the pyramidal cell layer thickness in the CA1, indicating significant neuronal loss (Fig. 2a) [8]. In the short term model, we find a strong increase of GFAP^+^ astrocytes cumulatively over the entire hippocampal CA1 subregion, with a trend of amassing in the stratum radiatum (SR) and stratum lacunosum moleculare (SLM) (Fig. 2a, a_1_). No changes were seen in the strata oriens (SO) and pyramidale (SP) (*data not shown*). When specifically assessing GLAST and S100β expression, the number of GLAST^+^, as well as S100β^+^ astrocytes (both a larger astrocyte cohort than GFAP), was not affected, possibly suggesting minor astrocyte activation (Figs. 2a_2_, S1b–b_2_) [28]. Similar to the above, the long-term paradigm presented a significant increase of GFAP-containing astrocytes in the SLM and SR (Fig. 3a, a_1_), but not the SO and SR (*data not shown*). Strikingly, a strong increase of GLAST^+^ and S100β^+^ astrocytes was found in the SR, with a trend in the SLM, without affecting layer thickness (Figs. 3a_2_, S1c–c_2_), indeed confirming that astrocytes accumulate in specific hippocampal layers after THC exposure.

To understand the layer-specific increase of astrocytes, we subsequently examined both proliferation and apoptosis. We first pulse-labelled dividing cells with EdU in vivo with coincident exposure to THC to reveal proliferation. We did not observe a significant proliferative response to THC exposure in either short-term or long-term paradigms (Figs. 2a_3_, 3a_3_, S2a, a_1_). We verified this by culturing primary hippocampal astrocytes with THC concentrations ranging from 10 nM to 1 µM, and confirmed a lack of astrocyte proliferation (Fig. S3), independent of growth-promoting serum concentrations (0%, 2% and 10% FBS). Second, we inspected acute cell death in the short-term paradigm with antibody labeling for cleaved caspase-3 (Fig. S2b, b_1_), but did not find any co-localization with both GFAP or GLAST::tdTomato, suggesting that neither survival nor proliferation of astrocytes is affected by exogenous THC exposure.

### Astrocytes Protect Neurons from THC-Induced Cell Death

**Fig. 4:**
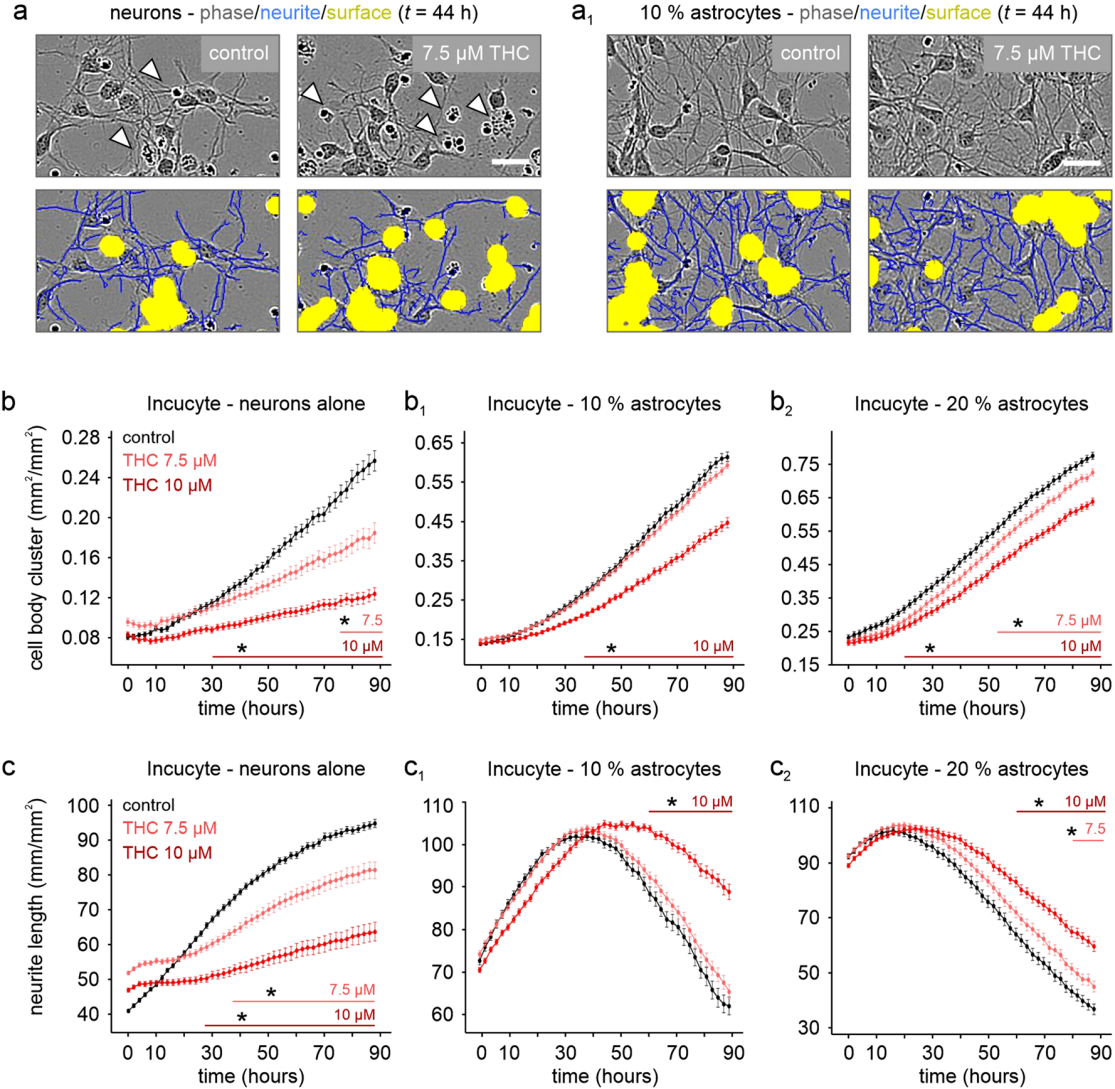
Astrocytes protect neurons from THC-induced cell death. **a** Phase contrast images at 44 h post THC application shows increased cell death (open arrows) and inhibition of growth/retraction of neurites (blue). Yellow denotes cell body area. **a**_**1**_ Low amounts of co-cultured astrocytes protect from cell death and neurite retraction. Note that the imaging software clusters cell bodies into large patches, inadvertently including some dead cells. In addition, neurite tracking loses resolution when high amounts of neurites are congregating into fiber bundles, as seen by a decrease in total neurite length after 30 h. **b**, **c** Live cell tracking of cell body clusters (**b**) and neurite outgrowth (**c**) without (**b**, **c**) and with co-cultured astrocytes (**b**_**1**_, **b**_**2**_, **c**_**1**_, **c**_**2**_). Scalebars = 25 µm (**a**, **a**_**1**_). ^+^P ≤ 0.1, *P ≤  0.05

We previously revealed a persistent reduction of CB1R^+^ neurons in hippocampal layers after preadolescent THC treatment in mice [8]. Since we did not see a complete neuronal loss in the CA1 pyramidal layer, and we find an increase of astrocytes (Fig. 3), we hypothesized that astrocytes could limit THC-induced neuronal death. This is significant since it could reconcile in vivo and in vitro data in the literature, with the latter demonstrating THC-induced cell death in neuron-specific cultures, but not brains [8]. We tested this by co-culturing primary hippocampal neurons with either low (10%) or high (20%) density of hippocampal astrocytes. By using closed-loop live cell imaging, we find that THC concentrations exceeding 5–7.5 µM reduced neuronal survival, as measured by cell body clusters per area, as early as 1 h past treatment (Fig. 4a), and was further impacted with 10 µM inducing near-complete loss after 24 h. However, neuronal survival was significantly rescued with the addition of 10% astrocytes already after 4 h, and almost entirely preserved in the presence of 20% astrocytes (Fig. 4a_1_–b_2_) at this time point. This effect was additionally reflected in the total neurite length per area after the first hour, where 10% astrocytes already significantly prevented THC-induced neurite retraction at 7.5 µM, while 20% astrocytes further improved neurite retraction at 10 µM exposure from two hours onwards (Fig. 4c–c_2_). Conversely, addition of astrocyte-conditioned medium alone to neuronal cultures did not prevent THC-induced cell damage (*data not shown*). These data indicate that either scavenging of stressors, or the physical presence of astrocytes, even in low quantities as compared to in vivo brain regions (with astrocyte-neuron ratios of 1.5 in the cerebral cortex) [29], is protective for neurons in the presence of micromolar concentrations of THC, which can be reached in rodent brains with a single cannabis preparation consumption [30].

### THC Affects Mitochondrial Metabolism

**Fig. 5:**
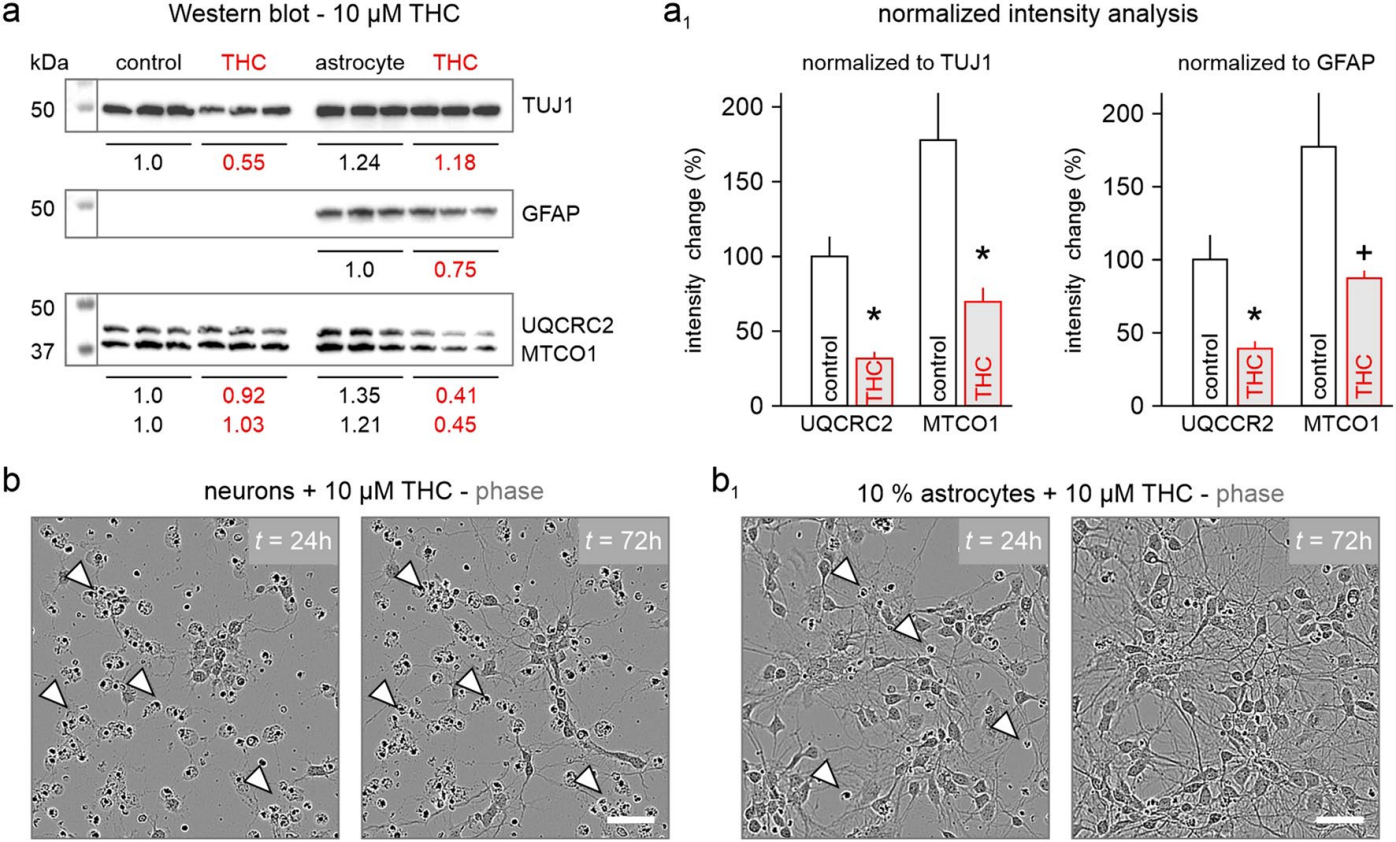
THC affects metabolic function by downregulation mitochondrial proteins. **a**, **a**_**1**_ Western blotting for cytoskeletal markers of neurons (TUJ1) and astrocytes (GFAP) in relation to mitochondrial proteins UQCRC2 and MTCO1. **b**, **b**_**1**_ Note that cellular debris remains firmly attached to cell culture dishes due to PDL, preventing accurate decreases in protein levels (open arrowheads). Scalebars = 50 µm (**b**, **b**_**1**_). ^+^P ≤ 0.1, *P ≤ 0.05

As we find CB1Rs expressed at low levels in astrocytes (Fig. 1), which are important for energy metabolism [8, 27], and EAAT2 is implicated in controlling metabolic rates [31], we probed if THC affects astrocyte survival and functioning. With high doses of THC, protein levels of TUJ1, a neuronal cytoskeletal marker, were significantly reduced in neuronal cultures (Figs. 5a, S4) in line with the above cell death observed (Fig. 4). We did not observe a complete loss of TUJ1 protein as some neuronal clusters maintained viability, and the fragmented somata and processes of dead cells remained firmly attached to the PDL-coated tissue culture dish up to 72 h post THC exposure (Fig. 5b). However, in the presence of 10% astrocytes, we detected a near complete rescue of neurons and TUJ1 expression (Fig. 5a), as well as an observed removal of cellular debris (Fig. 5b, b_1_). Strikingly, 10 µM THC reduced the expression of GFAP, indicating either a loss of astrocytes or inhibition of activity (Fig. 5a) [27, 28]. In line with this observation, there was a substantial decrease of Cytochrome b-c1 complex subunit 2 (UQCRC2; part of mitochondrial complex III), and mitochondrially encoded cytochrome c oxidase (MTCO1; the last enzyme of the mitochondrial electron transport chain), when normalized to both TUJ1 and GFAP. In sum, we find that postnatal THC exposure increases the presence of astrocytes in select hippocampal layers, irrespective of apoptosis or proliferation, suggestive of a protective function. Indeed, the physical presence of low numbers of astrocytes can protect neuronal survival and differentiation in the presence of micromolar concentrations of THC, even when astrocytes are metabolically impacted.

## Discussion

Our population is increasingly exposed to high amounts of THC due to the legalization and selective breeding of cannabis strains, and novel smoking routines that deliver the highest amount of THC possible in a short space of time [32]. As THC is transferred through the mother’s milk [33] and second hand smoke contains significant quantities of THC [34], young children are exposed to higher levels of THC as never seen before. Furthermore, due to the lipophilic nature of THC, it rapidly accumulates in tissues high in fat. Besides adipose tissue, the brain consist of up to 60% total fatty acid content [35], leading to concentrations of THC in the micromolar range [2], especially upon repeated exposure. This is of vital importance for infants and young adolescents, as cannabinoid receptor signaling is critical for neuronal migration, differentiation and circuit wiring [36–38] in virtually the whole brain. In particular, CB1Rs are highly expressed in the cortex and hippocampus [8, 39], making these structures particularly sensitive to the effects of THC. Thus, excess cannabinoid receptor signaling through accumulation of THC [40] can lead not only to receptor desensitization, mimicking a knockout phenotype [41], but also membrane destabilization through its perturbation of lipid bilayers [42], causing long-lasting changes in neuronal functioning.

The CA1 region of the hippocampus is one of the brain regions foremost susceptible to excitotoxicity, induced for instance by toxins, hypoxia, hypoglycemia or epileptic seizures [43, 44]. This is because its cyto-architectonic layered organization and cellular types limit plasticity by focal concentrations of glutamate at excitatory terminals. Under pathological conditions, excessive glutamate release from resident pyramidal neurons leads to a Ca^2+^ overload through NMDA receptors [45] and voltage-gated Ca^2+^ channels (noting their many subtypes expressed in pyramidal cells) [46]. Subsequently, cellular demise is propagated through the mitochondrion, which respond with the collapse of its membrane polarity and thus, ability to produce ATP as the terminal step of oxidative phosphorylation [47], resulting in the release of free oxygen radicals [48, 49] and subsequent activation of pro-apoptotic caspase cascades inducing cell death. This is why pharmacological agents that protect from membrane depolarization (e.g., Ca^2+^ channel blockers [43] (nimodipine, amlodipine), NMDA receptor inhibitors [44] or 5-HT1A receptor agonists [50, 51]) have profound positive effects on reducing the death of hippocampal neurons. Thus, the CA1 is uniquely sensitive to stressors inducing mitochondrial dysfunction and eventual resulting in neuronal apoptosis. Indeed, we previously demonstrated that exogenous THC exposure reduced mitochondrial functioning [8] of CB1R-containing excitatory pyramidal neurons and inhibitory interneurons in the CA1, inadvertently leading to neuronal death. In contrast to massive ischemic damage, we found that neurons were unevenly affected by THC, suggestive of a local neuroprotective effect limiting excessive cell damage.

Under pathological conditions [52], astrocytes are recruited to provide neuroprotective support by scavenging neurotransmitters including glutamate [53], as well as releasing adenosine [54] and glutathione [55] to limit metabolic and oxidative stress, respectively. As the scavenging of glutamate chiefly relies on EEATs [52] expressed by astrocytes, and we find its upregulation in our hippocampal proteomic dataset, we posited that the infiltration of astrocytes would protect the CA1 from excessive neuronal death upon THC exposure. Indeed, we find the mobilization of astrocytes in the strata lacunosum moleculare and radiatum indicative of a physiological response to pathological damage, similar to observations in neurodegenerative diseases such as epilepsy, ischemia and Alzheimer’s disease [14, 15, 52]. These thoughts are consistent with our co-culture experiments in which low density astrocytes (10%), in comparison to physiological numbers of 1.5 astrocyte-to-neurons in the cerebral cortex [29], were enough to significantly protect neurons from THC-induced damage. We firstly attribute the protective effect on the physical presence of astrocytes, providing *passive protection* by forming a larger lipid bilayer pool for THC to interact with [56], in addition to its possible capture into de novo created astrocytic lipid droplets in response to stressors [57, 58]. Second, even though astrocyte conditioned medium had no effect in preventing THC-induced damage, the close physical contact of neurons with astrocytes would allow for immediate local glutamate scavenging, as well as the release of high concentrations of ATP [59] and antioxidants [55], both independent of vesicular release [60, 61], directly onto the neuron, otherwise diluted in cell culture medium.

CB1R signaling has recently been demonstrated in intracellular organelles, including astrocytic mitochondria [27, 62], where mitochondrial CB1R downstream signaling results in decreased complex I activity and respiration, and subsequent modulation of neuronal energy metabolism [62]. In our co-culture experiments, we find a similar reduction in UQCRC2 (mitochondrial complex III), and MTCO1 (mitochondrial complex III, IV) protein levels, suggestive of decreased astrocytic activity. However, while these data are extensively collected by using THC and pharmacological tools, the contribution of the endogenous cannabinoid (endocannabinoid) signaling system under normal physiological circumstances is not understood. For instance, neither synthesizing nor degrading enzymes have been located to mitochondrial membranes, questioning if and where endocannabinoids are synthesized. Thus, to fully understand the effects of THC on cellular functioning, the focus should not only lay on the direct effects of THC, but also towards the understanding how it undermines physiological endocannabinoid signaling, necessary for a plethora of (non)-CB1R dependent functions [63].

## Materials and Methods

### Animals

Mice were kept under standard housing conditions on a 12-h/12-h light/dark cycle with food and water available ad libitum. Tissue collection and breeding of C57BL/6 and transgenic animals conformed to the 2010/63/EU directive and was approved by the Austrian Ministry of Science and Research (66.009/0145-WF/II/3b/2014 and 66.009/0277-WF/V3b/2017). Experimental protocols were constructed to reduce suffering as well as animal numbers.

### Drug Preparation and Applications

Synthetic (s)THC (Dronabinol, Gatt Koller), was diluted in dimethyl sulfoxide (DMSO, Sigma), vortexed and gently heated until fully dissolved. Aliquots and stock solution were stored at − 80 °C until further use. sTHC (5 mg/kg) was administered intraperitoneal (i.p) for 6 days from P9 to P14 and 2 days P9 to P10 in C57BL/6 and BAC-GLAST-Cre/ER::Ai14-tdTomato-loxP mice. For in vitro experiments, sTHC was diluted in DMSO and serial diluted into growth medium to prevent precipitation. To induce TdTomato expression, 4-hydroxytamoxifen (Sigma-Aldrich, 50 mg/kg) was dissolved in EtOH and stored at − 20 °C. At the day of injection, 4-OH tamoxifen stock was diluted with corn oil at 1:1 ratio and speed vacuumed at 45 °C for 1.5 h until EtOH was evaporated. For cell proliferation experiments, EdU (50 mg/kg) was dissolved in saline and injected at 10 mg/kg in vivo or 10 µM in vitro. Tamoxifen and EdU injections were performed once at the beginning of each experiment.

### Tissue Preparation

Animals were anesthetized with Isofluran and transcardially perfused with 1× PBS (0.1% Heparin, Sigma) for 4–5 min and subsequently perfused with a 4% paraformaldehyde (PFA) solution for 15 min with a dialysis pump (Per-Star Pro) adjusted to a speed flow of 1.5 ml per minute. Afterwards, brains were extracted and post-fixed in 4% PFA overnight shaking at 4 °C and cryoprotected with 30% sucrose solution, containing 0.1% sodium azide as a preservative, at 4 °C for at least 2 days. Brains were cryosectioned at 50 µm on a Thermo Scientific CryoStar NX70 and collected free floating in 1× PBS/azide.

### Immunohistochemistry

Prior to immunolabelling, non-specific binding was occluded with blocking solution containing 10% normal donkey serum (NDS, Jackson ImmunoResearch), 5% bovine serum albumin (BSA, Sigma-Aldrich) and 0.3% Triton X-100 (Sigma-Aldrich) in 0.1 M PB) for 2 h at RT whilst shaking. After blocking, the tissues were incubated with selected primary antibodies in 5% NDS, 0.2% BSA, 0.3% Triton X in 0.1 M PB for 48 h at 4 °C. The following antibodies were used: Rb-S100β (Synaptic Systems, 1:1000), Gp-GFAP (Synaptic Systems, 1:1000) and Ch-mCherry (EncorBio, 1:1000) for astrocytes; Rb-cleaved caspase 3 (Cell Signaling, 1:500) for apoptosis and hoechst 33,342 (Sigma Aldrich, 1:10,000) as nuclear counterstain.

After washing, tissues were incubated with Carbocyanine (Cy) conjugated secondary antibodies (Jackson Immuno Research, 1:300) diluted in 2% BSA in 1× PBS or 0.1 M PB for 2 h at room temperature. Secondary antibody solution was removed and sections washed 3–4 times for 30 min with 1× PBS or 0.1 M PB.

A Click-iT EdU Cell Proliferation Kit (ThermoFisher Scientific) was used to visualize EdU. In short, samples were treated with 0.5% Triton X-100 solution for 20 min, followed by three washing steps in PBS and subsequently 30 min at RT in the EdU reaction cocktail following the user guide lines. Sections were collected onto SuperFrost plus (Thermo Scientific) glass slides, dried overnight, and cover slipped with Entallan (Sigma-Aldrich) the next day.

### In Situ Hybridization

Brains of P3 and P8 mice were extracted, fresh frozen and sectioned with a CryoStar NX70 Cryostat at 16um. Sections were collected on SuperFrost Plus (Thermo Fisher Scientific) glass slides, air dried and stored at − 80 °C until use. Tissue sections were pre-treated with 20 min 4% PFA fixation at 4 °C, washed in PBS and dehydrated using increasing EtOH concentrations (25%, 50%, 75% 100%; 5 min each). In situ hybridization was performed for *Cnr1*, *Gfap* and *Glul* (glutamine synthetase) according to the HCR v3.0 protocol for “generic sample on the slide” (Molecular Instruments). Sections were imaged using an LSM 880 confocal microscope (Zeiss) and processed with the ZEN software (Zeiss).

### Primary Hippocampal Cultures

Primary neuronal cultures were obtained from E16.5 wild type mice hippocampi. Embryos were dissected in ice cold Hank’s balanced salt solution (HBSS) and their brains were diced and incubated in 0.1% Trypsin/DNase solution to enzymatically dissociate the tissue at 37 °C. Subsequently, trypsin was inhibited with DMEM/10% fetal bovine serum (FBS), and the cell suspension was further triturated with flamed glass Pasteur pipettes with decreasing bore sizes until a uniform cell suspension was achieved. Cell suspensions were layered on albumin-ovomucoid inhibitor and centrifuged at 70×*g* for 6 min to remove debris, resuspended and counted for plating. Neurons were plated on poly-d-lysine coated plates at a density of 50,000 cells per 96 well, and 500,000 cells per 6 well plate in Neurobasal A medium (Fisher Scientific), supplemented with 1× Glutamax (Thermo Fisher), penicillin–streptomycin (Gibco, 1%) and 2% B27 supplement (Gibco).

For astrocytes, P8 pups were dissected and their hippocampi were mechanically dissociated with flamed Pasteur pipettes. Cells were further enzymatically dissociated with 0.1% Trypsin/DNAse solution in DMEM, and treated with DMEM/10% FBS to inhibit trypsin. Astrocytes were plated in DMEM containing Glutamax, supplemented with penicillin–streptomycin (1%), sodium pyruvate (Gibco, 1%), FBS (10%), and N2 supplement (Gibco, 1%) in a T75 flask (NUNC). Medium was replaced after 24 h to remove excess cellular debris and grown to confluence (> 1 week) before being dissociated and plated out in complete Neurobasal A medium (see above). After plating, astrocytes were left to attach to the PDL covered surface (0, 10 or 20% of neuronal count), before neuronal cell suspensions were added. The following day, cells were treated with sTHC or vehicle (DMSO).

Cultures were imaged with an Incucyte live-imaging device (ZOOM and S3, Sartorius), using a 20× objective for up to 5 days. Cell cluster area, neurite length and neurite branching points were analysed over the entire time frame and plotted with its proprietary software (Sartorius).

### Western Blotting

sTHC (10 µM) treated co-cultures were lysed after 1 day of stimulation and homogenized in cell lysis buffer (100 mM Tris, pH 7.4, 150 mM NaCl, 1 mM EGTA, 1 mM EDTA, 1% Triton X-100, 0.5% sodium deoxycholate, 1× protease inhibitor, 1× phosphatase inhibitor) before preparing the total protein fractions. Protein concentrations were determined using the Pierce BCA Protein Assay Kit (Thermo Scientific). For protein analysis, 5.36 μg of each sample was used. Protein samples underwent a denaturation step at 95 °C for 5 min and loaded on 10% separation gels before being transferred onto a PVDF membrane. The membrane was blocked (5% skim milk powder (Sigma) in TBS-T) before primary antibodies were added for overnight incubation at 4 °C. The following antibodies were used: Ms-βIII-tubulin as neuronal marker (TUJ1, 1:3000), Gp-GFAP for glia (Synaptic Systems, 1:1000) and Ms-OXPHOS for mitochondrial subunits (Abcam, 1:500). Primary antibodies were removed through a series of TBS-T and TBS washes. Subsequently, membranes were exposed to HRP-conjugated secondary antibodies (Jackson Immuno Research, 1:1000) for 1 h at RT. Clarity Wester ECL Substrate (BioRad) was used for developing the HRP conjugated immunoblot and visualized with a ChemiDoc MP Imaging System (BioRad). Membranes were stripped with Restore Western blot stripping buffer (Thermo Scientific) and reimaged before restaining to prevent signal bleed through.

## Supplementary Information

Below is the link to the electronic supplementary material.

Supplementary file1 (JPG 339 kb)—**Figure S1.** Astrocyte numbers after THC exposure. (a, a_1_) Treatment paradigms used in this study. (b–c_2_) S100β cell counts in the CA1 subfield, SR and SLM in the short term (b–b_2_) and long term (c–c_2_) paradigm. *P ≤ 0.05.

Supplementary file2 (JPG 1718 kb)—**Figure S2.** Astrocyte proliferation and apoptosis in vivo. (a, a_1_) EdU stainings show no significant changes over hippocampal layers. Arrowheads denote astrocyte co-localization with EdU, while open arrowheads do not. (b–b_2_) Total numbers of cleaved caspase-3 levels in the CA1 subfield, SR and SLM of the short term paradigm. Scalebars = 100 µm (b_1_), 25 µm (a, b_1_ (right)). *P ≤ 0.05.

Supplementary file3 (JPG 761 kb)—**Figure S3.** Astrocyte proliferation in vitro. (a, a_1_) EdU stainings show no significant changes with GFAP co-labelling when cultured for 1 and 3 days in vitro (DIV). Arrowheads denote astrocyte co-localization with EdU. Scalebars = 10 µm.

Supplementary file4 (JPG 588 kb)—**Figure S4.** Western blot membranes. Full Western blot membranes used in Fig. 5.

## Data Availability

Enquiries about data availability should be directed to the authors.
