# Supplementary figures and images for: Resident Astrocytes can Limit Injury to Developing Hippocampal Neurons upon THC Exposure
Source: Neurochem Res. 2022 Dec 8;48(4):1242–53. doi: 10.1007/s11064-022-03836-1 (PMC10030412; doi:10.1007/s11064-022-03836-1)

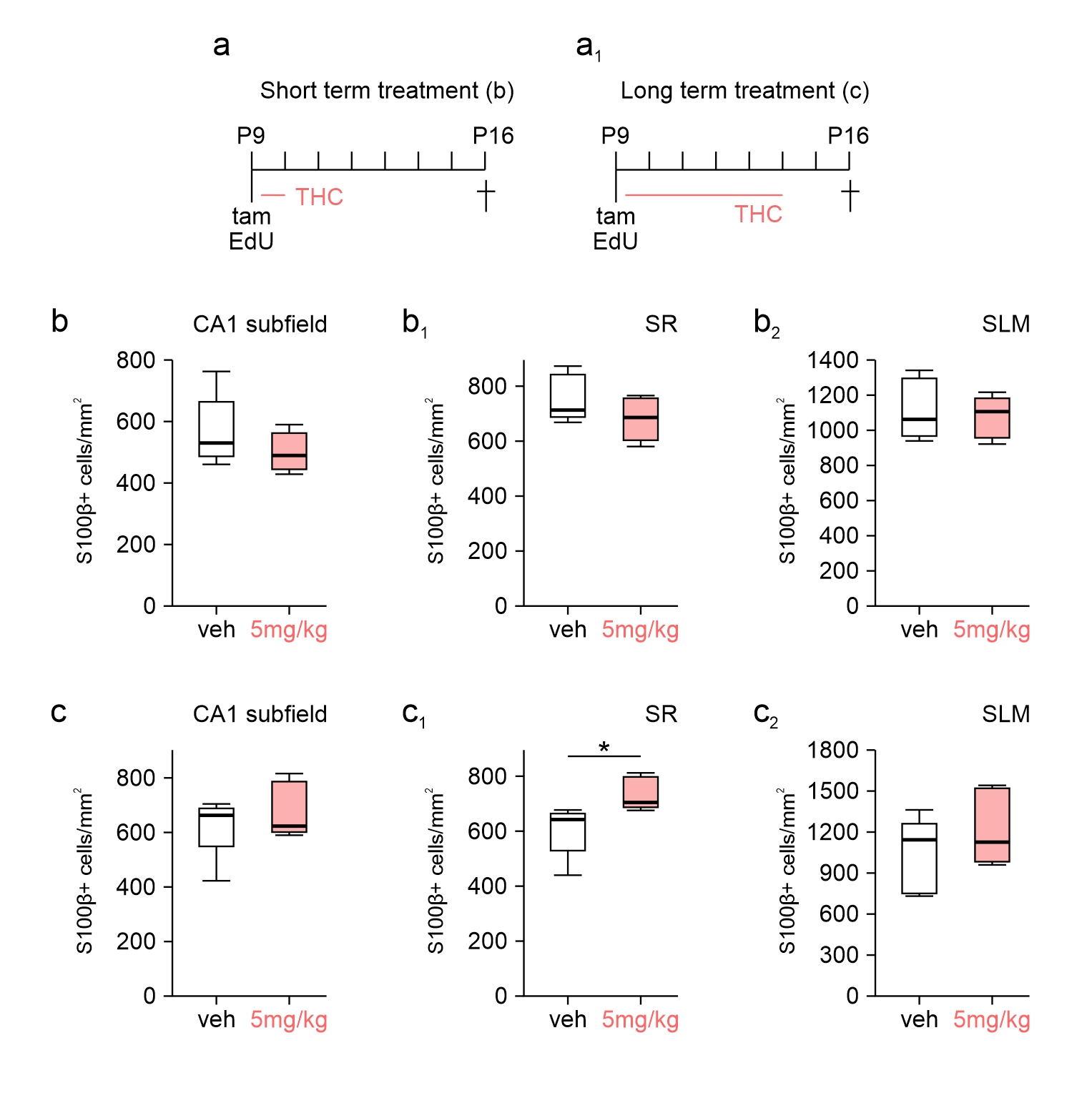

Supplement: Supplementary file 1 — Supplementary file1 (JPG 339 kb)—Figure S1. Astrocyte numbers after THC exposure. (a, a1) Treatment paradigms used in this study. (b–c2) S100β cell counts in the CA1 subfield, SR and SLM in the short term (b–b2) and long term (c–c2) paradigm. *P ≤ 0.05. [file 11064_2022_3836_MOESM1_ESM.jpg]

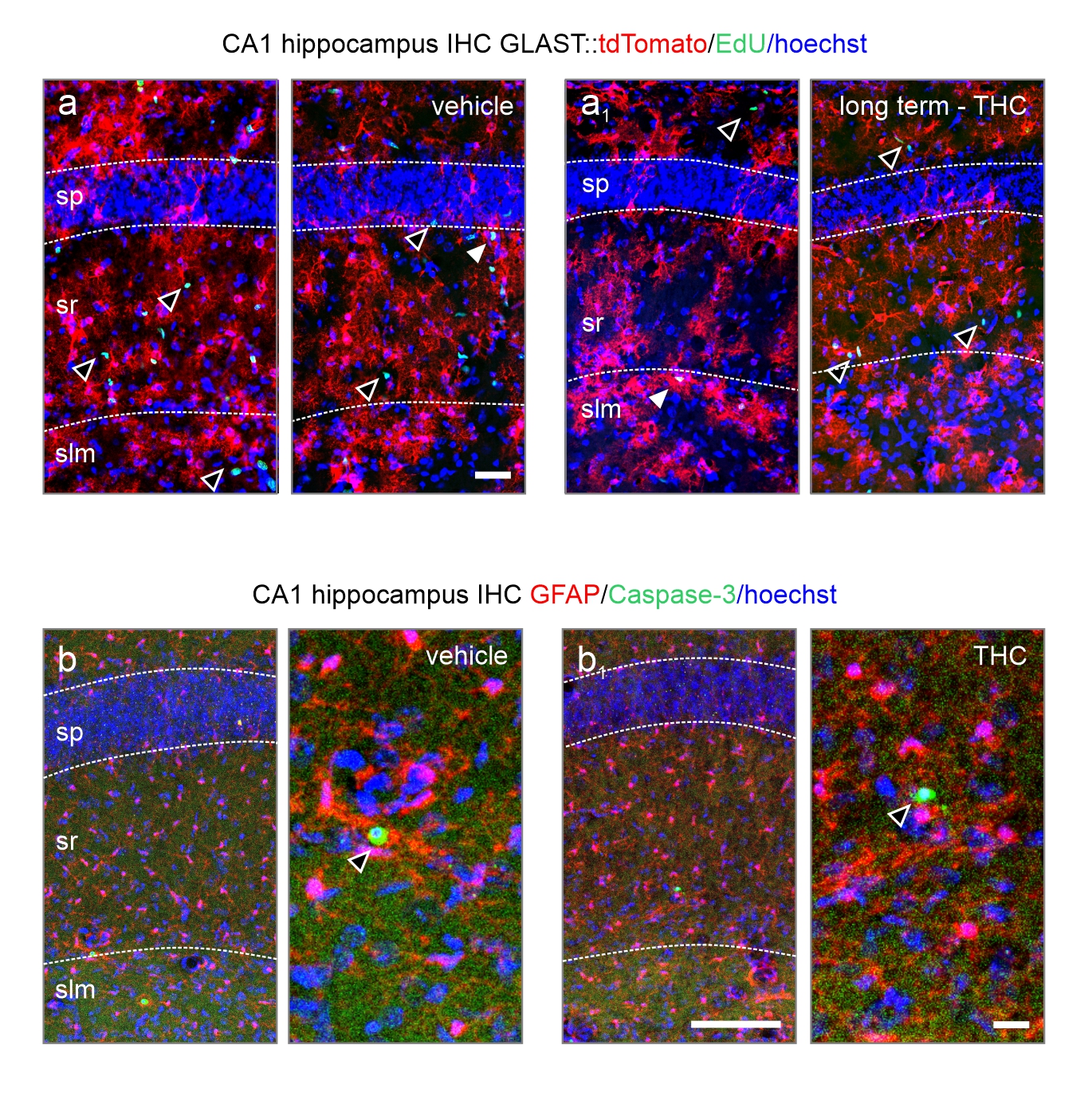

Supplement: Supplementary file 2 — Supplementary file2 (JPG 1718 kb)—Figure S2. Astrocyte proliferation and apoptosis in vivo. (a, a1) EdU stainings show no significant changes over hippocampal layers. Arrowheads denote astrocyte co-localization with EdU, while open arrowheads do not. (b–b2) Total numbers of cleaved caspase-3 levels in the CA1 subfield, SR and SLM of the short term paradigm. Scalebars = 100 µm (b1), 25 µm (a, b1 (right)). *P ≤ 0.05. [file 11064_2022_3836_MOESM2_ESM.jpg]

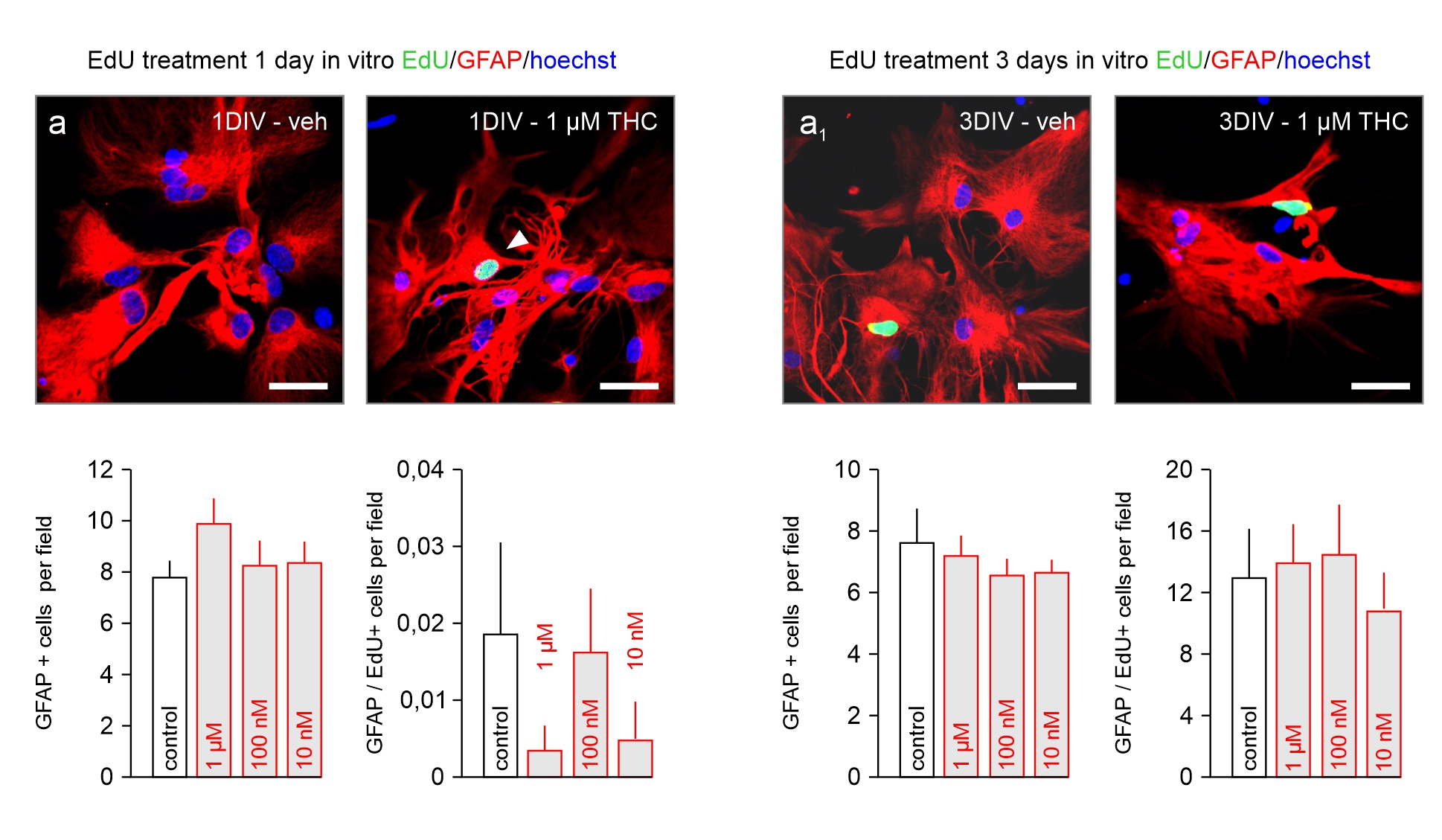

Supplement: Supplementary file 3 — Supplementary file3 (JPG 761 kb)—Figure S3. Astrocyte proliferation in vitro. (a, a1) EdU stainings show no significant changes with GFAP co-labelling when cultured for 1 and 3 days in vitro (DIV). Arrowheads denote astrocyte co-localization with EdU. Scalebars = 10 µm. [file 11064_2022_3836_MOESM3_ESM.jpg]

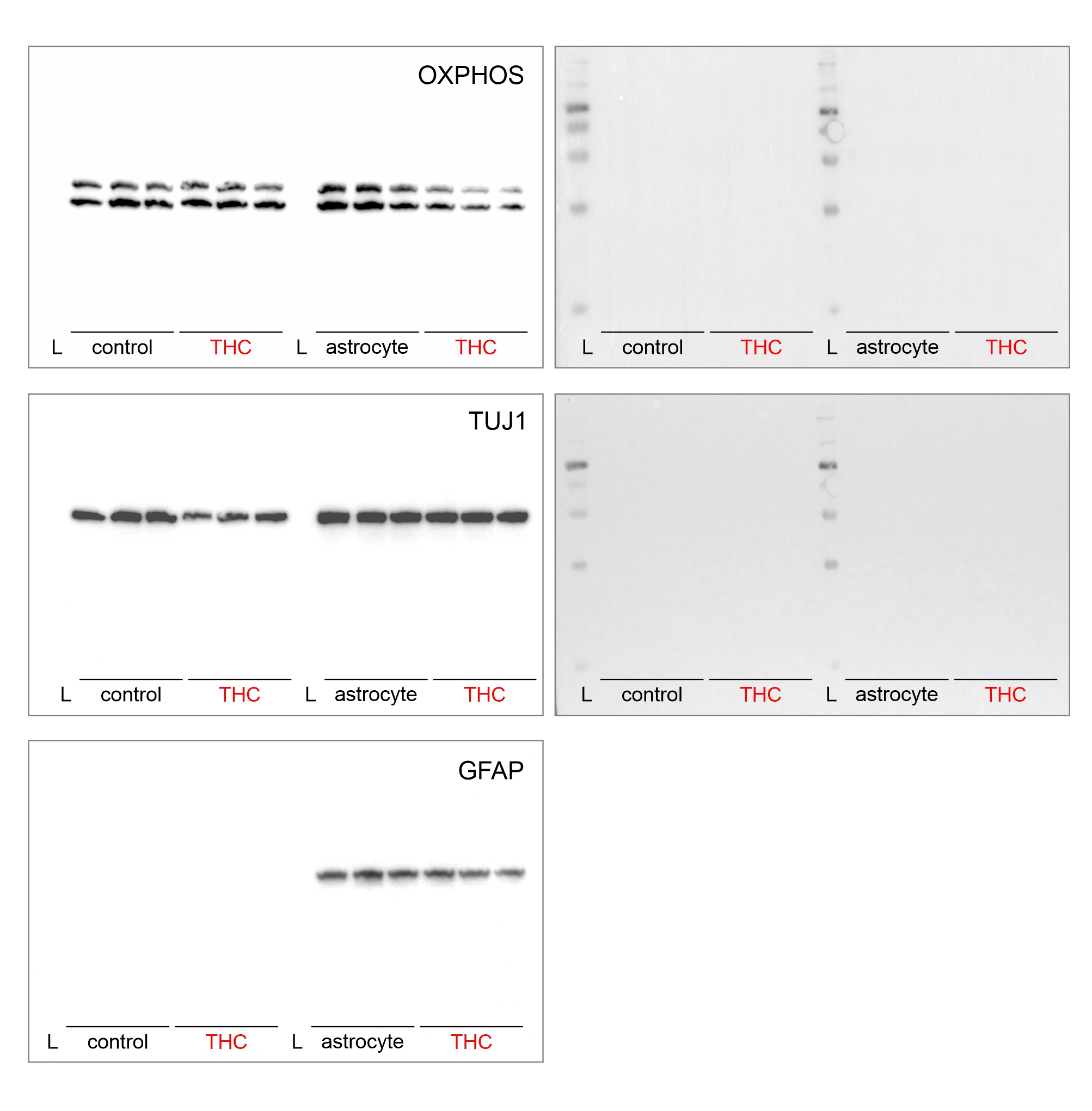

Supplement: Supplementary file 4 — Supplementary file4 (JPG 588 kb)—Figure S4. Western blot membranes. Full Western blot membranes used in Fig. 5. [file 11064_2022_3836_MOESM4_ESM.jpg]
